# Do we still need autopsy in times of modern multislice computed tomography?—Missed diagnoses in the emergency room

**DOI:** 10.1007/s00402-016-2588-4

**Published:** 2016-11-08

**Authors:** S. A. Euler, T. Kastenberger, R. Attal, M. Rieger, M. Blauth, M. Petri

**Affiliations:** 1 Department of Trauma Surgery, Medical University Innsbruck, Anichstr. 35, 6020 Innsbruck, Austria; 2 Department of Diagnostic & Interventional Radiology, Hall Regional Hospital, Milserstr. 10, 6060 Hall in Tirol, Austria; 3 Trauma Department, Hannover Medical School (MHH), Carl-Neuberg-Str. 1, 30625 Hannover, Germany

**Keywords:** Autopsy, CT scan, Emergency room, Missed diagnoses, Trauma

## Abstract

**Introduction:**

In spite of increasing quality of emergency room (ER) assessment in trauma patients and improved accuracy of modern multislice computed tomography (MSCT), the number of potentially missed diagnoses is still controversial. The aim of this study was to compare the initial findings of ER assessment and MSCT to the findings during autopsy in trauma patients not surviving the first 48 h after admission. We hypothesized that autopsy was more accurate than MSCT in diagnosing potentially fatal diagnoses.

**Patients and methods:**

Between January 2004 and September 2007, all trauma patients undergoing ER treatment in our institution who deceased within 48 h after admission were analyzed regarding diagnoses from initial ER assessment, including MSCT, and diagnoses from autopsy. Data were prospectively collected and retrospectively analyzed. Autopsy reports were compared to diagnoses of ER assessment and MSCT. Missed diagnoses (MD) and missed potentially fatal diagnoses (MPFD) were analyzed.

**Results:**

Seventy-three patients with a mean age of 53.2 years were included into the study. Sixty-three percent were male. Autopsy revealed at least one missed diagnosis in 25% of the patients, with the thoracic area accounting for 67% of these. At least one MPFD was found in 4.1% of the patients, all of them being located in the thorax. Total numbers of MD and MPFD were significantly lower for the newer CT generation (64 MSCT, *N* = 11), compared to older one (4 MSCT, *N* = 26).

**Conclusions:**

As determined by autopsy, modern multislice computed tomography is an accurate method to diagnose injuries. However, 25% of all diagnoses, and 4.1% of potentially fatal diagnoses are still missed in trauma patients, who deceased within the first 48 h after admission. Therefore, autopsy seems to be necessary to determine potentially missed diagnoses for both academic and medicolegal reasons as well as for quality control.

## Introduction

Emergency room (ER) management of trauma patients has been evolving. In addition to the traditional diagnostic means of X-ray and sonography, multislice computed tomography (MSCT) has been established in many trauma centers worldwide. MSCT is recommended in ER assessment of hemodynamically stable patients [1–3]. Recent studies have shown its diagnostic superiority compared to the conventional assessment, including radiography and sonography [1, 3]. In fatal cases, autopsy is still considered to be the gold standard for assessing the cause of death though for both academic and medicolegal reasons [4–9]. However, some authors have stated that autopsy after modern ER assessment is no longer required [10].

The aim of this study was to compare the findings of complete initial ER and MSCT assessment to the findings during autopsy in patients not surviving the first 48 h after admission. We hypothesized that autopsy was more accurate than MSCT in diagnosing potentially fatal diagnoses.

## Patients and methods

This study was approved by our institutional Ethical Committee. Between January 2004 and September 2007, all trauma patients undergoing ER treatment in our institution who deceased within 48 h after admission were analyzed regarding initial diagnoses from ER assessment including MSCT (including contrast medium), and diagnoses from autopsy. Two groups were defined due to an update of the CT scan to a newer generation in June 2006 (group 1: “generation 1 CT”: LightSpeed Qx/I, GE, Milwaukee, USA; group 2: “generation 2 CT”: GE LightSpeed VCT, GE, Milwaukee, USA). Patients who deceased prior to arrival in the ER, or who arrived under cardiopulmonary resuscitation (CPR), or who deceased more than 48 h after ER admission, were excluded. Only patients who received autopsy as well as complete standardized ER assessment, including complete MSCT scan of the head, neck, thorax, abdomen, pelvis, and obviously injured extremities, were included. If required, MSCT angiography for detection of vascular lesions, X-ray of the thorax following acute invasive interventions (central intravenous lines or drainages), and X-rays of injured extremities for pre-operative planning were added. All radiological diagnostics were performed by the particular senior radiologist on duty at the same time with the patient receiving the MSCT.

Data were prospectively collected and retrospectively analyzed. Autopsy reports were compared to diagnoses by ER assessment and MSCT, and missed diagnoses (MD) as well as missed potentially fatal diagnoses (MPFD) were analyzed. A senior expert radiologist analyzed whether MD and MPFD were caused by technical error, human error, or both.

Autopsy was performed in all patients by the department of Forensic Medicine or the department of Pathology of our institution. Autopsy findings were compared to the documented clinical data. The Injury Severity Score (ISS) was calculated for all patients. We primarily searched for MPFD, defined as injuries that raise the ISS and could independently have caused a fatal course of the patient. In patients with a MPFD, a new calculation of the adjusted ISS after autopsy was performed.

Statistic evaluation was performed using SPSS for Windows (SPSS, Chicago, Illinois). For specific indications, Wilcoxon test and for differences in arithmetic means and frequencies, Mann–Whitney, Chi-Quadrate, and Fisher’s exact tests were used. The level of significance was defined as *p* < 0.05.

## Results

In total, 1916 trauma patients that underwent ER treatment were evaluated. The overall mortality within the first 48 h after admission was 4.5%. In total, 73 patients with a mean age of 53.2 years were included into the study. Sixty-three percent were male.

Combining both groups, autopsy revealed at least one missed diagnosis in 25% of the patients, with the thoracic region accounting for 67% of these (Table 1). At least one MPFD was found in 4.1% of the patients, all of them being located in the thoracic region (2 aortic ruptures, 1 rupture of the inferior vena cava, Table 2). Total numbers of MD, and MPFD were significantly lower for group 2 (64 MSCT, *N* = 11), compared to group 1 (4 MSCT, *N* = 26) (Table 3). Total numbers of MD and MPFD for each group as well as the distribution of human/technical errors are displayed in Table 3.

**Table 1:** Detailed list of all missed diagnoses

Region	Number (%)	Group 1 (4 slice CT)	Group 2 (64 slice CT)
Head/neck/cervical spine [*N* = 6 (18.2%)]			
Fracture of the cranium	2	2	
Subarachnoidal bleeding	1		1
Hemorrhagic contusion of the cerebral cortex	1	1	
Avulsion of the ligamentary unit btw. skull base and cervical spine	1		1
Fracture of a cervical vertebra	1	1	
Thorax/thoracic spine [*N* = 22 (66.6%)]			
Fracture of ribs	6	4	2
Pulmonary fat embolism	4	4	
Aortic rupture	2	1	1
Rupture of the inferior vena cava	1	1	
Contusion of the heart	3	1	2
Rupture of the diaphragm	1	1	
Central avulsion of the pulmonary veins	1		1
Fracture of the sternum	1	1	
Luxation of the sterno-clavicular joint	1		1
Avulsion of a main bronchus	1	1	
Rupture of the pericardium	1	1	
Abdomen/lumbar spine [*N* = 5 (15.2%)]			
Rupture of the liver	4	3	1
Renal rupture	1	1	
Total	33

**Table 2:** Detailed summary of patients with MPFD (*N* = 3)

	Patient 1 (48 years; group 2; 64 slice CT)	Patient 2 (78 years; group 1; 4 slice CT)	Patient 3 (90 years; group 1; 4 slice CT)
Trauma	Blunt trauma (run over)	Blunt trauma (knocked down by a car)	Blunt trauma (run over)
Course	Hemodynamically unstable within ER Deceased after open thoracotomy and reanimation	Hemodynamically unstable within ER Deceased shortly after MSCT	Stable conditions at admission Severe deterioration within diagnostics Deceased shortly after MSCT
Diagnostics	Complete MSCT scan protocol	Complete MSCT scan protocol	Complete MSCT scan protocol
Diagnoses in ER	Multiple rib fractures Tension pneumothorax Bleeding from the right pulmonary artery Air in the right atrium and ventricle	Subdural and contusion hematoma, cerebral edema, midline shift Multiple rib fractures Fractures of clavicle and scapula Pneumothorax Pelvic fracture (Type B)	Subdural and subarachnoidal hematoma, midline shift Actively bleeding hematothorax Rupture of kidney Actively bleeding pelvic fracture (Type C)
Therapy	Chest drainage Massive blood transfusion Open thoracotomy and CPR	CPR	CPR
Missed potential fatal diagnoses	Incomplete rupture of aorta (loco typico) Central tear of pulmonary vein Rupture of Vena cava inferior Rupture of liver and kidney	Rupture of inferior vena cava Rupture of pericardium Rupture of liver	Rupture of descendent aorta

**Table 3:** MD and MPFD—nature of errors (absolute numbers; some patients did have two or more MD/MPFD, and human/technical errors occurred simultaneously in some cases)

	Overall	Group 1	Group 2	Overall technical error	Group 1 technical error	Group 2 technical error	Overall human error	Group 1 human error	Group 2 human error
MD	34	24	10	16	15	4	15	9	6
MPFD	3	2	1	3	2	1	2	1	1

There was no difference of ISS between the status after ER and MSCT assessment (median ISS 24) and the adjusted status following autopsy (median ISS 25).

## Discussion

The most important finding of this study was that multislice computed tomography missed at least one diagnosis in 25%, and at least one potentially fatal diagnosis in 4.1%, as determined by autopsy in trauma patients not surviving the first 48 h after admission. 67% of the missed diagnoses and 100% of the potentially fatal diagnoses were located in the thoracic region. Total numbers of MD and MPFD were significantly lower for the newer CT generation (64 MSCT, *N* = 11), compared to older one (4 MSCT, *N* = 26). The time period of 48 h until decease of the patient as inclusion criterion for the study was chosen to exclude secondary potentially fatal complications, such as pneumonia, SIRS, sepsis, organ dysfunction or MODS as possible confounding factors.

Our demographic data were in accordance with the previous reported cohorts. Fung Kon Jin et al. quote a number of 16 patients in one year (trauma, autopsy, at least partial diagnostic assessment but variable times of death after admission) [11]. Sharma et al. included 160 patients in four years (trauma, autopsy, death within 24 h, at least partial diagnostics but also patients admitted under CPR or already dead) [7]. The average age (53.16 years) and distribution of gender (63% male) of our cohort also matches the values of comparable studies [4, 11, 12].

In our cohort, 4.4% of all trauma patients admitted over the ER did not survive the first 48 h in hospital. Compared to previous studies, this is a quite low value. Matthes et al. found an early lethality within the first 24 h of 8.7% in severely injured patients [13]. Other authors found an early lethality of 46 through 61% within the first 48 h [4, 7, 10, 12]. Differences in definitions used for “trauma” among the various studies consequently lead to different selection criteria for eligible patients.

The incidence of all missed diagnoses in our study was 25%. In other studies, the incidence ranges from 0% (Forsythe et al. [10]) to 57.6% (Sharma et al. [7]). Different autopsy rates in many studies, differences in quality, regulations of accuracy and requested exploration may be reasons for the discrepancies. Furthermore, the definition of a missed diagnosis varies. Many authors only included severe MD or MPFD [7, 10]. The MPFD rate in this study was 4.1%. Reported rates of MPFD range from 0 to 14% [4, 7, 8, 12].

Injuries of the thorax represented the area with the majority of MD (66.6%), followed by head/neck (18.2%), and abdomen/pelvis/extremities (15.2%). This corresponds well to quotes in prior studies [4, 12].

Secondary complications and comorbidities were not counted as MD. During autopsy, venous thrombosis of the sinus was found in five patients as a diagnosis not known before. This diagnosis was valued as secondary complication and thus not counted as a MD. Nevertheless, diagnoses like bronchopneumonia and severe haemorrhage are dedicated to be the most common MD in trauma patients [4, 7].

Reasons for missing a diagnosis by means of MSCT scan are numerous, including a variety of human and technical errors. While human errors appear to be more or less constant and independent from the resolution of the CT scanner, technical errors appear to be reducible by sufficient image thickness and image intervals. In the particular setting of hemodynamically unstable patients, insufficient circulation patterns may compromise the distribution of injected contrast medium and accordingly the detection of active bleeding injuries.

As for the survey of the ISS, severities of traumas were reflected in the identified values. After autopsy, we did not find a significant change of the ISS (median ISS 24 before, and 25 after autopsy). Hodgson et al. reports an increase from 30 to 43 in trauma patients after autopsy [8]. Other authors describe an increased ISS after autopsy in 7–69% of all patients [12, 14].

The question whether MSCT is accurate enough to replace autopsy is of particular interest. In 2007, Molina et al. conducted a study on 113 trauma patients who obtained a CT scan within 24 h before death and who finally underwent autopsy [15]. They found unacceptably low rates of sensitivity and specificity as well as positive and negative predictive values for MSCT. This study distinctly challenges the idea of post mortem CT diagnostics, particularly in the forensic area. However, it has to be considered that this study was conducted between 2002 and 2005, using CT scanners with image thickness of 5–10 mm at 5-to-10-mm intervals for the skull, 3-mm image thickness at a 2-mm interval for the cervical spine, and 5-mm image thickness at a 5-mm interval for the chest, abdomen, and pelvis. This makes their findings comparable to our group 1 and illustrates the impact of high-resolution CT scans with sufficient slice thickness and slice intervals on diagnostic accuracy. Our results suggest that the rate of technical errors can be reduced by using a 64 MSCT scanner compared to the older 4 MSCT scanner.

This study has several limitations. First, only missed diagnoses offering a direct relation to the causative trauma were considered. Second, MSCT scan was done in some patients with insufficient circulation patterns, which made it impossible to exactly evaluate the distribution of injected contrast medium and accordingly to detect all active bleeding injuries. Third, due to the retrospective study design and partially insufficient documentation—e.g., imaging from external referring hospitals or lost records, we were not able to fully report all missed diagnoses on all patients. 32 patients had to be excluded for this reason.

## Conclusions

As determined by autopsy, modern multislice computed tomography is an accurate method to diagnose injuries. However, 25% of all diagnoses, and 4.1% of potentially fatal diagnoses are still missed in trauma patients, who deceased within the first 48 h after admission. Therefore, autopsy seems to be necessary to determine potentially missed diagnoses for both academic and medicolegal reasons as well as for quality control.

## References

[1] Huber-Wagner S, Lefering R, Qvick LM, Körner M, Kay MV, Pfeifer KJ et al. (2009) Effect of whole-body CT during trauma resuscitation on survival: a retrospective, multicentre study. Lancet 25 373(9673):1455–1461 10.1016/S0140-6736(09)60232-4 19321199

[2] Rieger M, Czermak B, El Attal R, Sumann G, Jaschke W, Freund M (2009). Initial clinical experience with a 64-MDCT whole-body scanner in an emergency department: better time management and diagnostic quality?. J Trauma.

[3] Rieger M, Sparr H, Esterhammer R, Fink C, Bale R, Czermak B (2002). Modern CT diagnosis of acute thoracic and abdominal trauma. Anaesthesist.

[4] Barendregt WB, de Boer HH, Kubat K (1993). Quality control in fatally injured patients: the value of the necropsy. Eur J Surg.

[5] Goldman L, Sayson R, Robbins S, Cohn LH, Bettmann M, Weisberg M (1983). The value of the autopsy in three medical eras. N Engl J Med.

[6] Hodgson NF, Stewart TC, Girotti MJ (2000). Autopsies and death certification in deaths due to blunt trauma: what are we missing?. Can J Surg.

[7] Sharma BR, Gupta M, Harish D, Singh VP (2005). Missed diagnoses in trauma patients vis-a-vis significance of autopsy. Injury.

[8] Stothert JC, Gbaanador GB, Herndon DN (1990). The role of autopsy in death resulting from trauma. J Trauma.

[9] Steinwall D, Befrits F, Naidoo SR, Hardcastle T, Eriksson A, Muckart DJ (2012). Deaths at a Level 1 Trauma Unit: a clinical finding and post-mortem correlation study. Injury.

[10] Forsythe RM, Livingston DH, Lavery RF, Mosenthal AC, Hauser CJ (2002). Autopsies in trauma do not add to peer review or quality assurance. J Trauma.

[11] Fung Kon Jin PH, Klaver JF, Maes A, Ponsen KJ, Das C, Goslings JC (2008). Autopsies following death due to traumatic injuries in The Netherlands: an evaluation of current practice. Injury.

[12] Ong AW, Cohn SM, Cohn KA, Jaramillo DH, Parbhu R, McKenney MG (2002). Unexpected findings in trauma patients dying in the intensive care unit: results of 153 consecutive autopsies. J Am Coll Surg.

[13] Matthes G, Seifert J, Ostermann PA, Wurfel S, Ekkernkamp A, Wich M (2001). Early death of the severly injured patient—a retrospective analysis. Zentralbl Chir.

[14] Streat SJ, Civil ID (1990). Injury scaling at autopsy: the comparison with premortem clinical data. Accid Anal Prev.

[15] Molina DK, Nichols JJ, Dimaio VJ (2007). The sensitivity of computed tomography (CT) scans in detecting trauma: are CT scans reliable enough for courtroom testimony?. J Trauma.

